# Supplementary material for: On-surface preparation of coordinated lanthanide-transition-metal clusters
Source: Nat Commun. 2021 Mar 12;12:1619. doi: 10.1038/s41467-021-21911-z (PMC7954866; doi:10.1038/s41467-021-21911-z)
Supplement: Supplementary file 1 — Supplementary Information [file 41467_2021_21911_MOESM1_ESM.pdf]

Supplementary Information for

On-Surface Preparation of Coordinated Lanthanide-Transition-Metal Clusters

Jing Liu[1,2], *Jie Li*[1,3], *Zhen Xu*[1], *Xiong Zhou*[4], *Qiang Xue*[1], *Tianhao Wu*[1], *Mingjun Zhong*[1], *Ruoning Li*[1], *Rong Sun*[4], *Ziyong Shen*[1], *Hao Tang*[5], *Song Gao*[2,4,6], *Bingwu Wang*[4], *Shimin Hou*[1,3], *Yongfeng Wang**[,1,2,6]

[1]Key Laboratory for the Physics and Chemistry of Nanodevices and Center for Carbon-based Electronics, Department of Electronics, Peking University, Beijing 100871, China

[2]Division of Quantum State of Matter, Beijing Academy of Quantum Information Sciences, Beijing 100193, China

[3]Peking University Information Technology Institute (Tianjin Binhai), Tianjin 300450, China.

[4]Beijing National Laboratory of Molecular Science, College of Chemistry and Molecular Engineering, Peking University, Beijing 100871, China

[5]CEMES, UPR CNRS 8011, 29 Rue Jeanne Marvig, 31055 Toulouse Cedex 4, France

[6]Institute of Spin Science and Technology, South China University of Technology, Guangzhou 510641, China

E-mail: yongfengwang@pku.edu.cn

SUPPLEMENTARY FIGURES

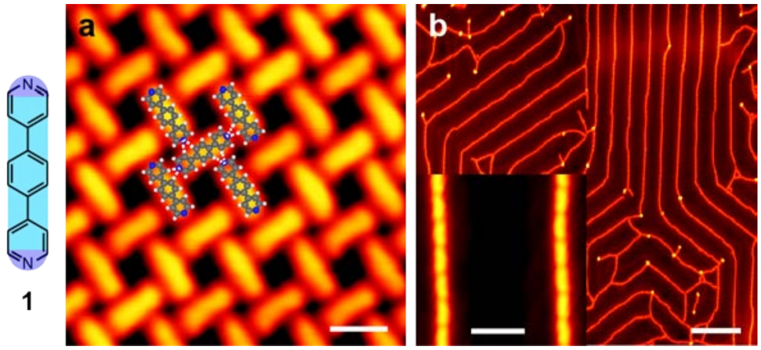

Supplementary Figure 1. Structures formed by **1** without Ce on Au(111). STM images of (a) the self-assembly of **1** on Au(111) (scanning conditions: bias *V* = 10 mV, tunneling current *I* = 80 pA, imaging temperature *T* = 77 K) with the molecular models superimposed and (b) the 1D coordinated molecular wires formed by **1** and Au adatoms on Au(111) (*V* = 100 mV, *I* = 50 pA, *T* = 4.3 K). The intermolecular hydrogen bonds in the self-assembled structure are illustrated by the white dashed lines in (a). Scale bars: (a) 1 nm, (b) 15 nm, inset: 3 nm.

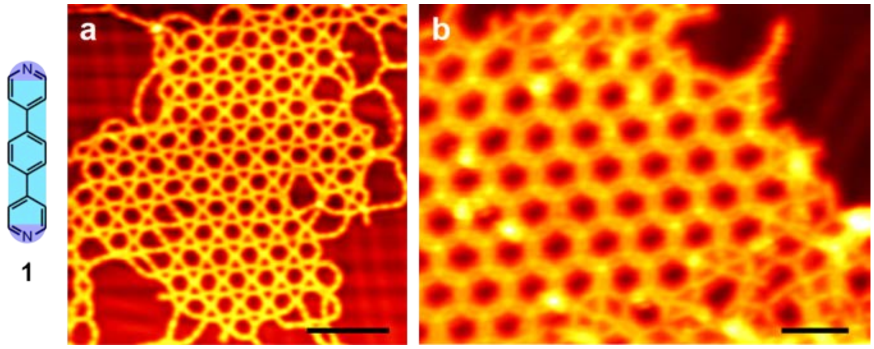

Supplementary Figure 2. Coordination structures involving Ce and **1** formed on Au(111). Large-area STM images of (a) the Kagome network (*V* = 200 mV, *I* = 20 pA, *T* = 77 K) and (b) the double-wall honeycomb structure (*V* = -100 mV, *I* = -20 pA, *T* = 77 K) formed by depositing Ce and **1** on Au(111). Scale bars: (a) 10 nm, (b) 5 nm.

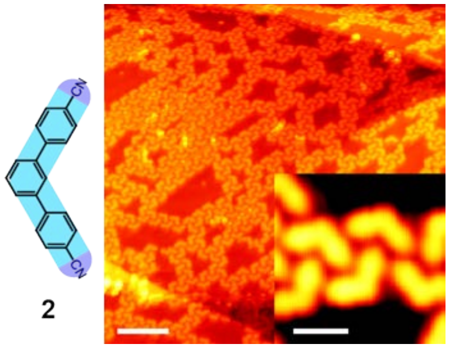

Supplementary Figure 3. STM image of the hydrogen-bond-stabilized braid-like self-assemblies of **2** on Au(111) (V = 1 V, I = 20 pA, T = 4.3 K; V = 10 mV, I = 20 pA, T = 4.3 K for inset). Scale bars: 5 nm, inset: 1 nm.

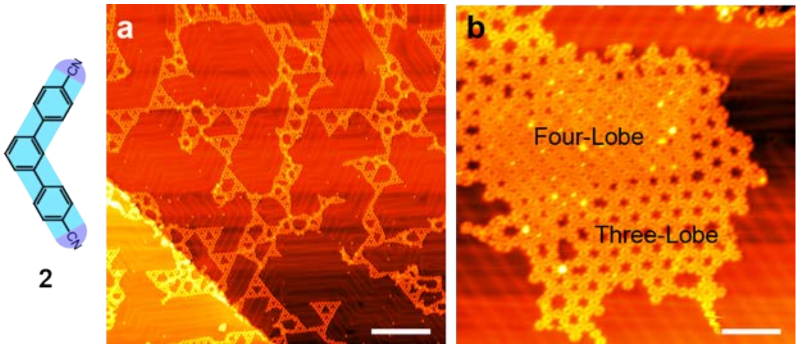

Supplementary Figure 4. Coordination structures involving Ce and **2** formed on Au(111). Large-area STM images of (a) the Sierpiński triangles (V = 100 mV, I = 20 pA, T = 77 K) and (b) the three-lobe and four-lobe structures (V = 100 mV, I = 20 pA, T = 4.3 K) formed by depositing Ce and **2** on Au(111). Scale bars: (a) 30 nm, (b) 10 nm.

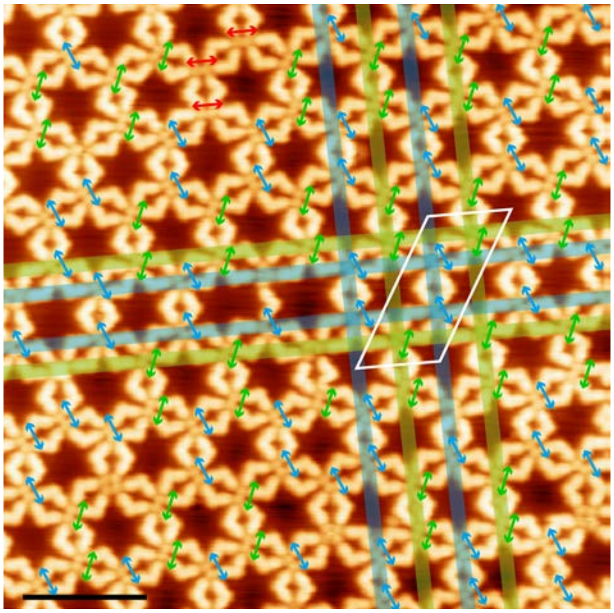

Supplementary Figure 5. STM image of the three-lobe structure formed by depositing **2** and Ce on Au(111) (V = 10 mV, I = 20 pA, T = 4.3 K) with the orientations of the Ce dimers marked by the arrows. The unit cell of the network is shown by the white parallelogram. Green and blue lines are superimposed to highlight the alignment of the Ce dimers with the same orientation. Scale bar: 5 nm.

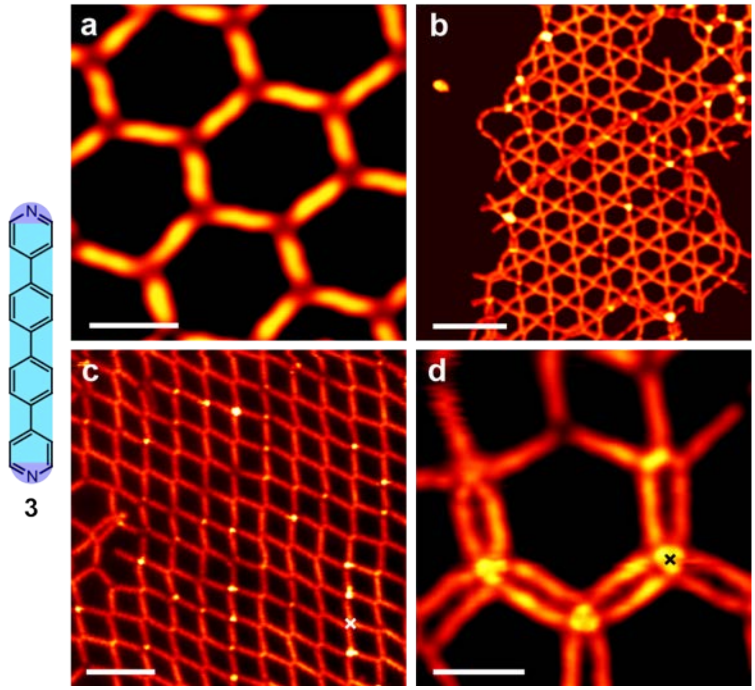

Supplementary Figure 6. Coordination structures involving Ce and **3** formed on Au(111). STM images of (a) the single-wall honeycomb network (V = 100 mV, I = 20 pA, T = 77 K), (b) the Kagome structure (V = 100 mV, I = 20 pA, T = 77 K), (c) the fishing-net structure (in constant-height mode, V = 100 mV, I = 50 pA at the point marked by the cross, T = 77 K) and (d) the coordination motifs comprising the three-Ce-containing clusters (in constant-height mode, V = 100 mV, I = 50 pA at the point marked by the cross, T = 77 K) formed by depositing Ce and **3** on Au(111). Scale bars: (a) 2 nm, (b) 10 nm, (c) 5 nm, (d) 2 nm.

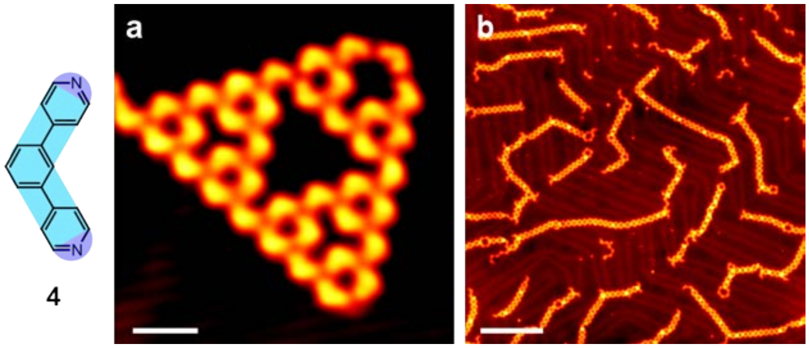

Supplementary Figure 7. Coordination structures involving Ce and **4** formed on Au(111). STM images of (a) a quasi-3rd-order Sierpiński triangle (V = -50 mV, I = -20 pA, T = 4.3 K) and (b) the chain-like structures (V = -100 mV, I = -20 pA, T = 4.3 K) formed by depositing Ce and **4** on Au(111). Scale bars: (a) 2 nm, (b) 15 nm.

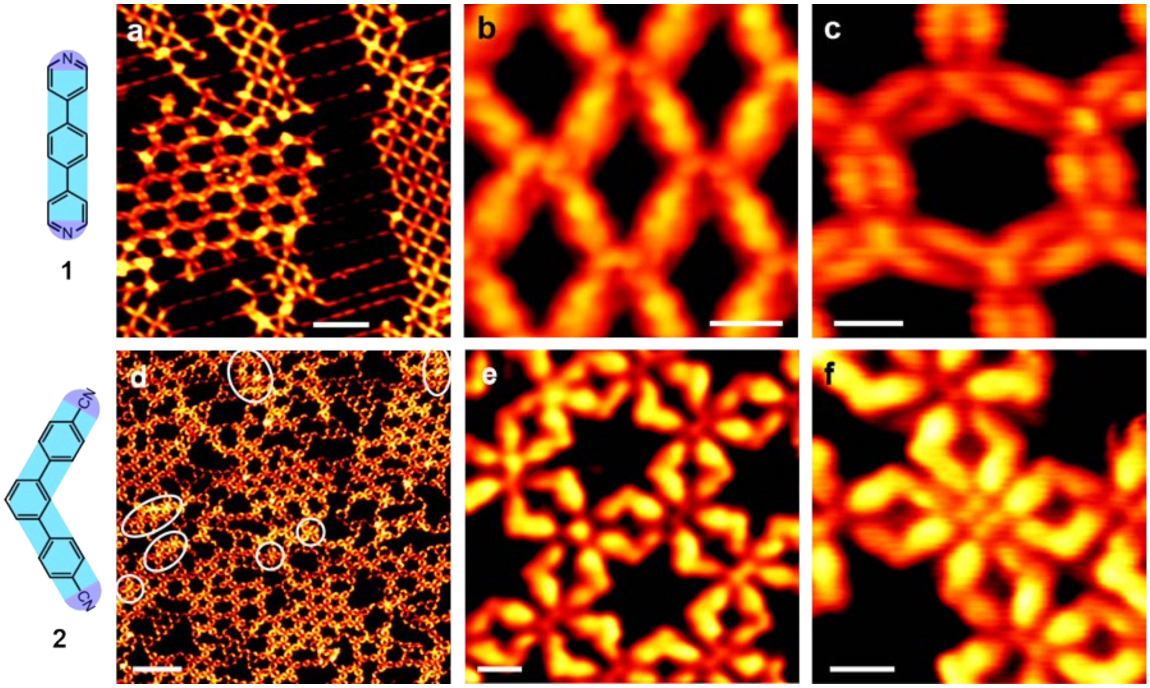

Supplementary Figure 8. Coordination structures involving Ce and **1** or **2** formed on Au(100). (a) Large-area STM image of the coexisting fishing-net networks and double-wall honeycomb structures formed by depositing Ce and **1** on Au(100) (*V* = 100 mV, *I* = 20 pA, *T* = 77 K). Magnified STM images of (b) the fishing-net network (*V* = 5 mV, *I* = 100 pA, *T* = 77 K) and (c) the double-wall honeycomb structure (*V* = 100 mV, *I* = 20 pA, *T* = 77 K). (d) Large-area STM image of the coexisting Sierpiński triangles, three-lobe networks and four-lobe motifs formed by depositing Ce and **2** on Au(100) (*V* = 100 mV, *I* = 20 pA, *T* = 77 K). The four-lobe motifs are highlighted by the white circles. Magnified STM images of (e) the three-lobe structure (*V* = 100 mV, *I* = 20 pA, *T* = 77 K) and (f) the four-lobe structure (*V* = 1 mV, *I* = 20 pA, *T* = 77 K). Scale bars: (a) 5 nm, (b, c) 1 nm, (d) 10 nm, (e, f) 1 nm.

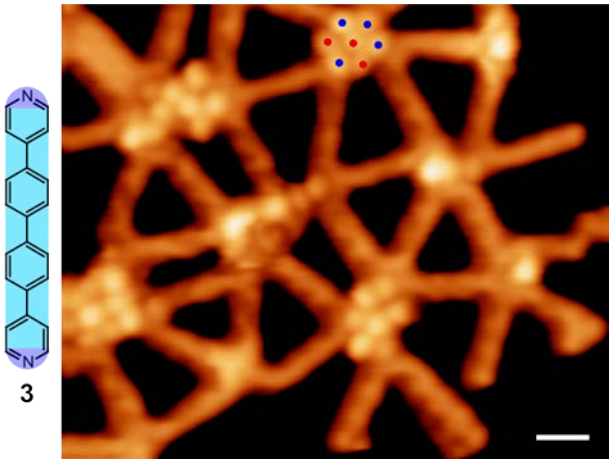

3

Supplementary Figure 9. STM image of the less-ordered multinuclear coordination structure formed by **3** and Ce on Au(111) (V = 3 mV, I = 50 pA, T = 77 K). Examples of the Ce atoms coordinated with the molecular ligands (Ce_L) and those surrounded by the other Ce atoms (Ce_{Ce}) are marked by blue and red dots, respectively. Scale bar: 1 nm.

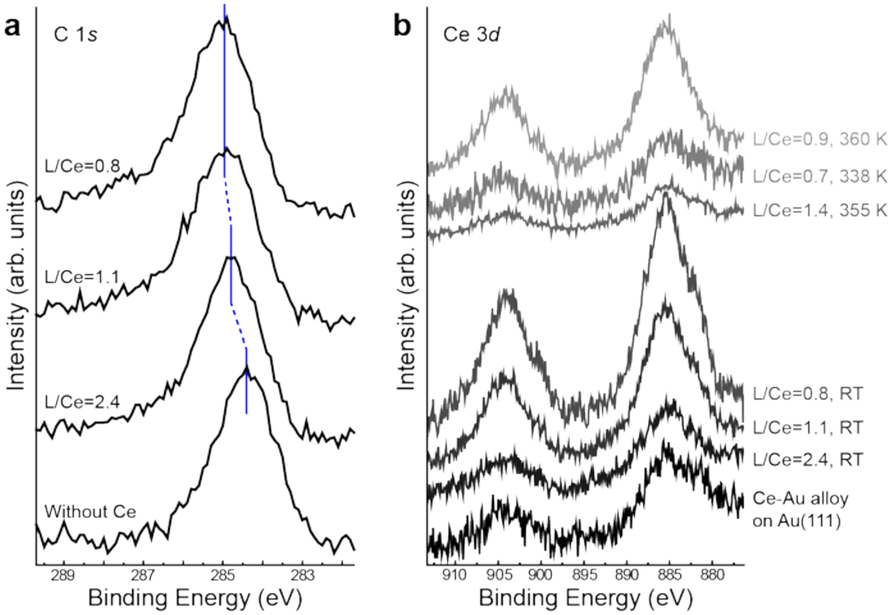

Supplementary Figure 10. XPS results of the samples with Ce and ligand **2** on Au(111). (a) C 1*s* spectra of the samples with different molecule-to-Ce (L/Ce) ratios obtained after annealing the samples at room temperature (RT). (b) Ce 3*d* spectra collected by varying the molecule-to-Ce ratios and annealing temperatures of the samples. The sample of Ce-Au alloy on Au(111) was prepared by depositing Ce onto the Au(111) substrate at RT following Supplementary Refs. 1 and 2.

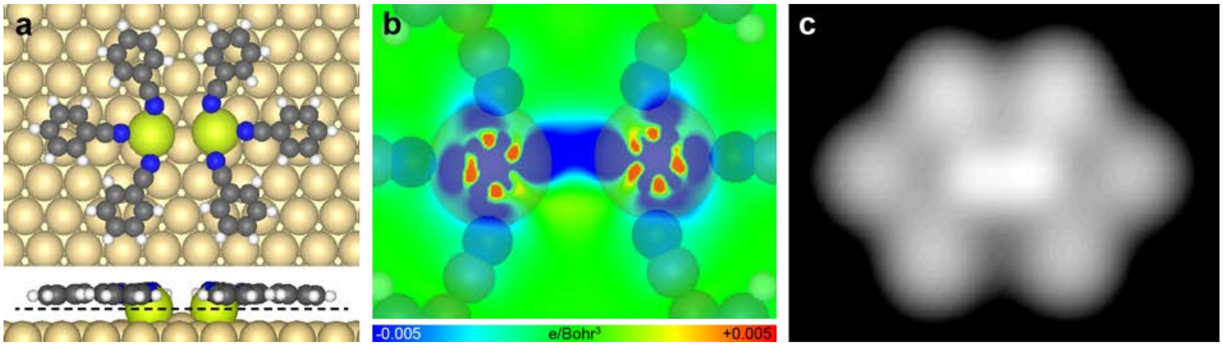

Supplementary Figure 11. Theoretical results of the $Ce_2(CN)_6$ structure. (a) Optimized model (upper: top view, lower: side view), (b) cross section of the differential electron density along the black dashed line in (a), and (c) simulated STM image of the $Ce_2(CN)_6$ motif on Au(111).

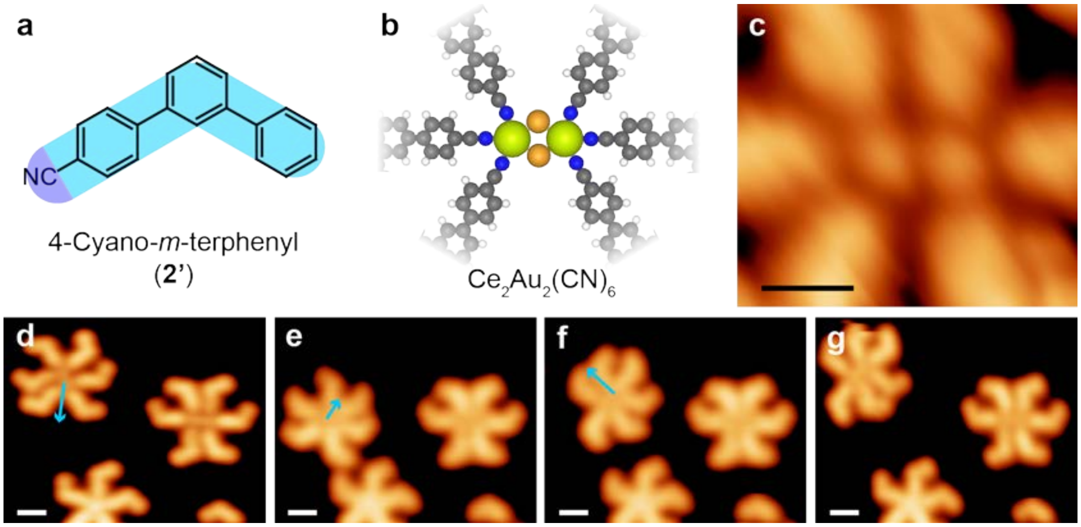

Supplementary Figure 12. Tip-manipulation experiments. (a) Chemical structure of 4-cyano-*m*-terphenyl (**2'**). (b) Schematic model of the $Ce_2Au_2(CN)_6$ motif. (c) STM image of the two-Ce-containing center of the coordinated supramolecular structure formed by **2'** and Ce on Au(111) (V = 10 mV, I = 200 pA, T = 4.3 K). (d-g) STM images of the same area showing the lateral displacements of the coordinated supramolecule by the sequential tip-manipulations (V = 10 mV, I = 120, 30, 50, 120 pA, respectively, T = 4.3 K). The blue arrows mark the paths of the tip. Scale bars: (c) 0.5 nm, (d-g) 1 nm.

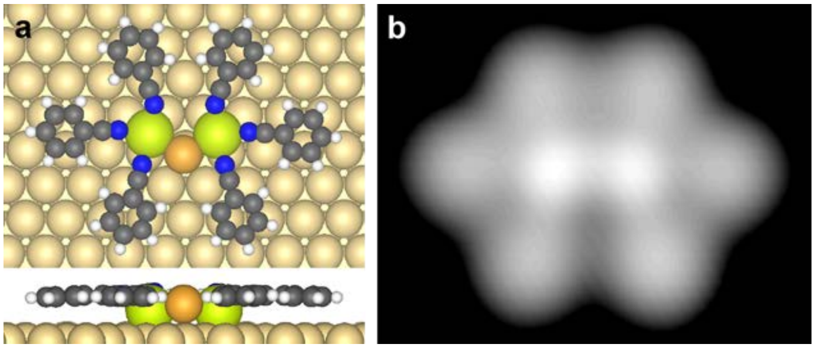

Supplementary Figure 13. Theoretical results of the $Ce_2Au(CN)_6$ structure. (a) Optimized model (upper: top view, lower: side view) and (b) simulated STM image of the $Ce_2Au(CN)_6$ motif on Au(111).

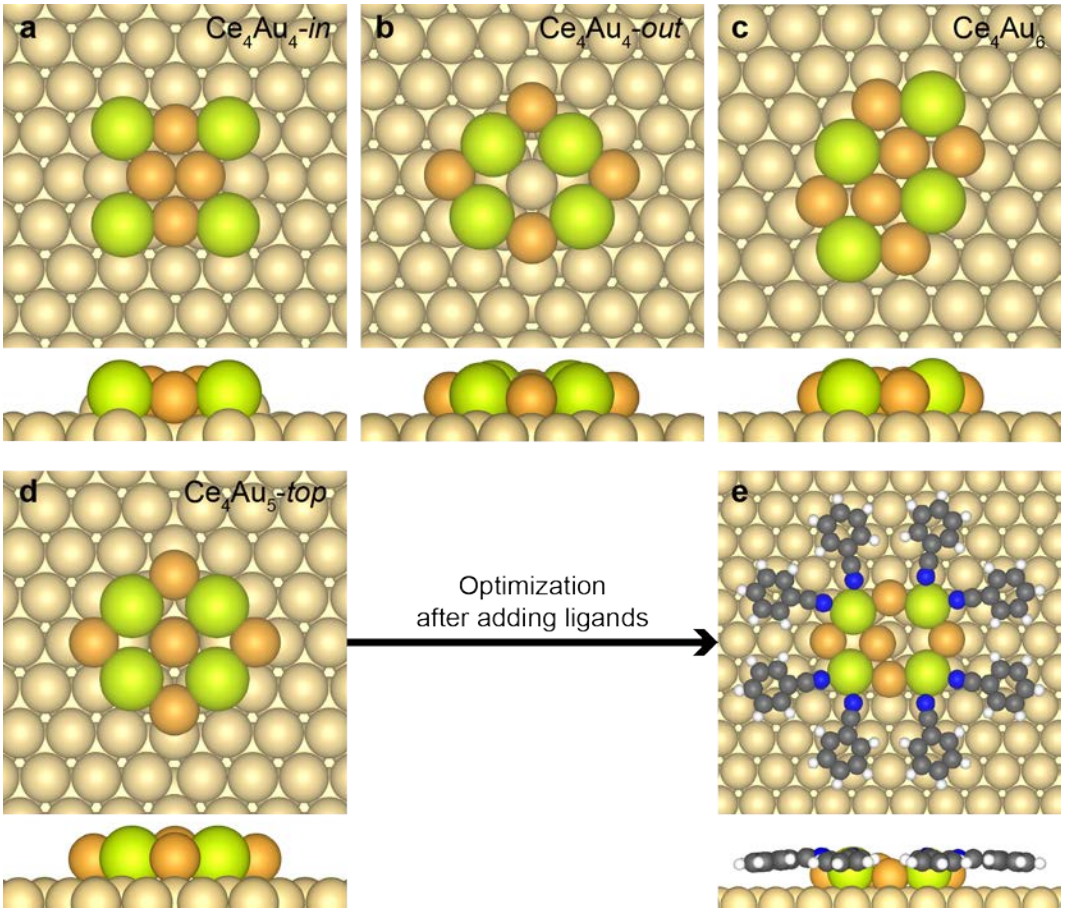

Supplementary Figure 14. Theoretically tested models of the four-Ce-containing cluster. Optimized models of (a) the Ce_4Au_4-*in* cluster, (b) the Ce_4Au_4-*out* cluster, (c) the Ce_4Au_6 cluster, (d) the Ce_4Au_5-*top* cluster, and (e) the $Ce_4Au_6(CN)_8$ motif constructed based on the Ce_4Au_5-*top* cluster on Au(111) (upper: top view, lower: side view).

SUPPLEMENTARY DISCUSSION

Tip-manipulation experiments.

4-Cyano-*m*-terphenyl (denoted as **2'**, Supplementary Fig. 12a) was employed as the ligand to construct coordination structures with Ce atoms on Au(111). With the same molecular backbone as ligand **2** but only one nitrile group, ligand **2'** coordinated with Ce in the same way as that found for the three-lobe network formed by **2** and Ce, i.e., to form $Ce_2Au_2(CN)_6$ motifs (Supplementary Fig. 12b) as demonstrated later in the main text, giving rise to the isolated supramolecular structures as presented in Supplementary Fig. 12c,d. The formation of the isolated supramolecules facilitates the lateral tip-manipulation experiment. As a result, sequential lateral manipulations of a coordinated supramolecule comprising a two-Ce-containing cluster were achieved as shown in Supplementary Fig. 12d-g.

Theoretically tested models of the coordinated four-Ce-containing cluster.

For the coordination structure comprising Ce tetramers, i.e., the four-lobe network, in addition to the $Ce_4Au_5(CN)_8$ motif as shown in Fig. 6c in the main text, several other structures were also tested. Firstly, three candidate models of the Ce-Au cluster other than Ce_4Au_5 were tested without taking the molecular ligands into account. They are two Ce_4Au_4 clusters in which the four Au adatoms are located at either the inner (denoted as Ce_4Au_4-*in*) or the outer (denoted as Ce_4Au_4-*out*) side of the four Ce atoms, and a Ce_4Au_6 cluster. The optimized model of Ce_4Au_4-*in* (Supplementary Fig. 14a) shows a Ce-Ce distance of 5.66 A, which is larger than the experimentally measured value (4.9 ± 0.5 A). The Ce atoms in the optimized models of Ce_4Au_4-*out* (Supplementary Fig. 14b) and Ce_4Au_6 (Supplementary Fig. 14c) are arranged in the different symmetries from the experimentally observed square-shaped Ce tetramers. As a consequence, all

the three structures were ruled out due to their poor consistency with the experimental results. Moreover, the Ce_4Au_5 cluster located at a different position with respect to the substrate from that presented in the main text (Fig. 6c) was also considered (denoted as Ce_4Au_5-*top* since the central Au adatom of the cluster was located at the top site of the substrate in this model). The optimized model of the Ce_4Au_5-*top* cluster is shown in Supplementary Fig. 14d. The further optimization of the coordination structure constructed by adding eight nitrile ligands to the optimized Ce_4Au_5-*top* cluster resulted in the distortion of the metallic center (Supplementary Fig. 14e), which disagrees with the experimental observations. Therefore, the Ce_4Au_5-*top*-based coordination motif was also excluded as the building block of the four-lobe network.

SUPPLEMENTARY REFERENCES

1. Ma, S., Zhao, X., Rodriguez, J. A. & Hrbek, J. STM and XPS study of growth of Ce on Au(111). *J. Phys. Chem. C* **111**, 3685-3691 (2007).
2. Zhao, X., Ma, S., Hrbek, J. & Rodriguez, J. A. Reaction of water with Ce-Au(111) and CeO_x/Au(111) surfaces: Photoemission and STM studies. *Surf. Sci.* **601**, 2445-2452 (2007).
